# A qualitative exploration of owner experiences following dog adoption

**DOI:** 10.1017/awf.2025.4

**Published:** 2025-02-10

**Authors:** Bethany Joy Moyer, Helen Zulch, Beth Ann Ventura, Oliver Burman

**Affiliations:** Animal Behaviour, Cognition, and Welfare Research Group, School of Natural Sciences, University of Lincoln, Brayford Pool Campus, Lincoln LN6 7DL, UK

**Keywords:** adjustment, animal shelter, animal welfare, behaviour, dog, rescue

## Abstract

The adjustment period — wherein adopted animals transition to their new home — is a critical time for animal welfare and owner satisfaction, yet literature varies in estimates of how long this period lasts in dogs. This study sought to better characterise the adjustment period in relation to owner experience and canine welfare and clarify its duration as perceived by owners. We used a qualitative approach to examine owner perceptions of duration and their experience of the adjustment period. Twenty-seven interviews were conducted and analysed using thematic content analysis. Six themes were described: adjustment period duration; behavioural indicators during adjustment; behaviours indicative of adjustment; factors influencing adjustment; owner change in routine; and adjustment concerns. Over half of participants perceived the adjustment period to last longer than four months. Behaviours used by owners to assess dog adjustment included: moderation of behavioural extremes (e.g. lethargy and restlessness); play; tail wagging; greeting; and learning their routine. Owners reported that prior experiences and medical needs impacted the adjustment duration, that they adjusted aspects of their lifestyle, and raised concerns about being the right fit for their dog. Future research should incorporate a longer time-frame to better understand how and when dogs adjust to a home, and as there is variation in dog behaviour during adjustment, it must also account for individual differences. As we develop a better understanding of how to characterise this period, adopters can be better prepared for the initial months of dog ownership, and interventions can be individualised to improve owner experience and dog welfare.

## Introduction

Approximately 11.5 million dogs (Murray *et al.* 2015) are kept as pets in the UK, with an estimated 14% of pet owners in the UK acquiring their dog from a rescue organisation (People’s Dispensary for Sick Animals [PDSA] 2022). Of these, a proportion of adopters struggle to help their pet settle into their new home and thus re-relinquish their dogs. For example, Diesel *et al.* (2008) reported that approximately 14% of shelter dogs in the UK were returned within six months of adoption, which aligns with previous research that likelihood of relinquishment decreases over time, with this risk remaining elevated for the first year and decreasing following two years (New *et al.* 2000). These estimates suggest a better understanding of the adjustment period — and what might be done to promote easier and/or faster adaptation — is needed to improve welfare of dogs transitioning from the shelter to a home environment. The adjustment period can be described as the time in which an animal adapts to their new environment, routine and owner (Johnstone 2021). However, the length of the adjustment period, and factors that influence it, are currently unclear. Adoption literature (i.e. literature distributed for adopters and rescue organisations that is not itself scientific or peer-reviewed literature) has varyingly indicated that this process can range from four to six weeks (Wolfe 2019) or up to three months (Aldred 2022); but, to our knowledge, these estimates are not supported through scientific evidence. In contrast, scientific literature on shelter dogs has found that, following adoption, physiological measures of stress such as cortisol levels may change for up to six months following adoption (van der Laan *et al.* 2022), suggesting that the adjustment period may extend at least up to six months. Similarly, research on (non-rescue) dogs has indicated that physiological markers of stress following transition to a kennel environment, an event that likely reflects some of the changes experienced during rehoming, may be present for upwards of ten weeks (Rooney *et al.* 2007). Thumpkin *et al.* (2024) also found evidence that adopters felt it took between three to six months for dogs to trust their owners, further indicating the fact that this period may extend beyond the time-frame in adoption literature.

Long-term sheltering can have behavioural and physiological impacts on dogs that can reflect diminished welfare (Dalla Villa *et al.* 2013), including elevated stress levels, decreased immune function and increased stereotypic behaviour (Protopopova 2016). Dogs within a kennelled environment tend to have higher cortisol levels than those in a home environment (Rooney *et al.* 2007). Behavioural interventions can have beneficial impacts on stays in the shelter (Protopopova & Gunter 2017), especially when considering short-term impacts. Enrichment (Herron *et al.* 2014), human contact (Normando *et al.* 2009), and short-term fostering can reduce physiological and behavioural indicators of stress and so appear to improve animal welfare (Gunter *et al.* 2019). However, the use of interventions, particularly training, to alter adoption rates has had mixed results, with some studies finding that the improved behaviour is beneficial for increasing adoption rates (d’Angelo *et al.* 2022), and other studies finding no effect compared to a control group (Protopopova & Gunter 2017).

Prior adoption research has focused extensively on factors that influence adoption, pet retention, and behaviour problems both within and outside the rescue shelter. For example, morphological characteristics (e.g. breed, size, coat colour) impact how quickly dogs are adopted from a rescue shelter (Diesel *et al.* 2008). Kennel behaviour can also have an impact on the time it takes for a dog to be adopted, with locomotion, leaning on walls, and facing backward apparently extending the length of stay of shelter dogs, while barking, jumping, and sitting did not have an impact (Protopopova *et al.* 2014). Similarly, research has indicated that adopters prefer dogs who lie down near the potential owner and engage in play rather than dogs who are too active or inattentive (Protopopova & Wynne 2014). Adopter expectations also play a role in owners’ satisfaction and the risk of return of recently adopted pets, with returning owners being more likely to expect that their dog was not fearful in new situations, was friendly towards children, responsive to training, and would not dig/chew inappropriately, as well as having higher expectations for the dog-owner bond (Powell *et al.* 2022). Research has indicated the benefits of behavioural counselling on improving dog behaviour and/or adopter satisfaction; dog adopters who received pre-adoption counselling on house-training were more likely to perceive their dog’s house-training as successful (Herron *et al.* 2007). In addition, these owners were also more likely to use enzymatic cleaners and less likely to use verbal punishment, following the recommendations given in the pre-adoption training (Herron *et al.* 2007). Herron *et al.* (2014) also found that while pre-adoption counselling did not prevent the occurrence of separation anxiety in adopted shelter dogs, owners who received counselling were more likely to follow the recommendation of leaving their dogs with food or a toy, indicating some implementation of the counselling suggestions. Similarly, Gazzano *et al.* (2008) found that counselling from a veterinary behaviourist was beneficial in preventing problematic behaviours in puppies.

In addition to studies showing the physiological impact of adoption and switching environments, a recent study used the C-BARQ (Canine Behavioral Assessment and Research Questionnaire) to track behavioural change throughout the six months following adoption (Bohland *et al.* 2023). Notably, ‘excitability’ and ‘touch sensitivity’ increased between three and six months, while ‘stranger-directed aggression’, ‘chasing behaviour’, and ‘training difficulty’ increased over the six months. Meanwhile, ‘separation-related behaviours’, ‘attachment’ and ‘attention-seeking behaviours’ decreased across the duration of the six-month post-adoption period (Bohland *et al.* 2023).

However, individual tools may not provide a complete picture of adjustment. While the C-BARQ is used for a variety of purposes, its primary purpose is to measure behavioural problems (Serpell undated), so positive or non-problematic behavioural changes may be missed. Furthermore, the adjustment process likely encompasses a variety of factors apart from behavioural changes, for example, development of the dog-owner relationship bond and eventual reduction of physiological stress. As a result, the C-BARQ may not comprehensively reflect the owner experience. Bohland *et al.* (2023) noted that many participants believed that their dog’s behaviour improved, despite the C-BARQ showing that behaviour worsened in several categories. It may be that owners perhaps do not consider all behaviours as equally problematic, or that owners may consider behaviours that are not reflected in the C-BARQ as being important to improving behaviour. Finally, the C-BARQ was used to investigate changes at a population level, rather than individually, so differences between dogs in how behaviour may change following adoption may not be identified.

Focusing on individual dog outcomes over time is important to characterise and provide insights into the adjustment process. Concurrently, a deeper understanding of human factors that interact with this adjustment process is needed to identify areas that impact people during the adoption process. Understanding adopters’ experiences from their perspective can complement understanding of the adjustment period as new owners are the ones living with and best positioned to affect (and be affected by) their new dog. In turn, this may also help improve adoption outcomes as key time-points and needs for additional support throughout the adjustment process may be identified.

A qualitative approach allows for a deeper understanding of human experiences (Jones 1995), and has previously been used to examine owners’ perceptions of dog acquisition (Holland *et al.* 2021), changes to dog behaviour during COVID-19 lockdowns (Boardman & Farnworth 2022), and owner motivations for dog walking (Westgarth *et al.* 2017). However, to our knowledge, there have been few studies directed at understanding the experiences of new dog owners around this period (e.g. Thumpkin *et al.* 2024). Therefore, our study used a qualitative semi-structured interview approach to explore common experiences of shelter dog adopters with respect to how dogs adjust to a new home. We sought to establish a timeline of the dog adjustment period and to identify any commonly reported changes in dog behaviour, aspects of the adopter’s home environment and human-dog interactions that may contribute to the perceived length of the adjustment period, as well as how smoothly this progresses from the adopter’s perspective.

## Materials and methods

### Study approval

This study was approved by the University of Lincoln (ref: UOL 2023_12690).

### Participant recruitment

Prospective participants were recruited via convenience sampling by advertising on fourteen social media groups in February and March 2023, including dog-focused interest groups and groups related to survey research recruitment. Interested individuals were directed to complete a screening survey to ensure they met the inclusion criteria for the study as follows: participants must have been over the age of eighteen years of age; residing in the UK; and have, within the last two years, adopted a dog from a kennel-based rescue or shelter within the UK, a foster-based organisation within the UK, or organisations abroad that either transported rescue dogs to the UK or adopted out rescue dogs to UK residents. All dogs had to have resided in the home for a minimum of three months prior to the interview occurring. If a participant had more than one dog meeting the study criteria, they were asked to focus on the dog whose name came first alphabetically.

### Interviews

Semi-structured interviews were chosen as they incorporate open-ended questions that allow for individuals to elaborate on their experiences and give the interviewer flexibility to follow up on participant answers (Adams 2015). This approach allowed for participants to drive the conversation around their relevant experiences (Ritter *et al.* 2023). Semi-structured interviews and a qualitative approach are also both useful for exploring areas that are not heavily researched (Adams 2015) which, due to the limited research on post-adoption adjustment, was beneficial for this study.

In order to refine question clarity, the interview guide (see Supplementary material) was initially piloted with three individuals who owned rescue dogs but who did not match inclusion criteria and whose results are therefore not reported. The final interview guide consisted of 17 key questions and prompted participants to share what they remembered about the days, weeks, and months shortly after adopting their dog, their relationship with their dog, their dog’s behaviour, their dog’s personality, and the support they had received following adoption. Terms such as ‘adjustment’ or ‘settling’ were not defined for participants. Whilst we acknowledge that this could lead to inconsistencies between participants based on how they interpreted these terms, this allowed them to bring their own perspectives to the interview.

Questions followed the same structure for each interviewee (i.e. the same questions asked in the same order), with follow-up questions to allow any clarifications if necessary. Online interviews were chosen to support researcher access and allow more flexibility for participants, as they mitigate the need to travel and make it possible for people outside of the researcher’s geographic area to participate (Mirick & Wladkowski 2019).

During data collection, BM, HZ, and OB discussed the ideas and potential themes that became apparent during the interview process. Interviews were scheduled until the authors felt that saturation — when new concepts and themes were not appearing in later interviews (Glaser & Strauss 1967) — had been reached, based on the reoccurrence of topics in interviews. The authors felt that saturation was reached after the completion of 27 interviews and thus no further interviews were scheduled.

### Data analysis

Interviews were recorded and auto-transcribed by MS Teams (Microsoft®, Redmond, WA, USA) and transcriptions checked and edited for accuracy by BM. During this process, BM read and re-read the transcripts to become familiar with the data. Following transcription and prior to analysis, transcripts were member-checked, where participants had an opportunity to read their transcript to make edits and ensure that it accurately reflected their experience (Creswell & Miller 2000).

The full transcripts were analysed with NVivo12 by BM using a thematic content analysis (Elo & Kyngäs 2008), a qualitative research analysis method that organises participant responses into common codes and themes. This process began by BM first familiarising herself with the data during the interview and transcription process, and then coding the data using an inductive process, in which the set of codes was created using the data from the transcripts (Elo & Kyngäs 2008). Initially, transcripts were annotated, and the annotations were used to create the first set of codes. Transcripts were reviewed multiple times during the analysis as codes were created, changed, and condensed to allow the researcher to identify patterns and themes within the transcripts. Since the interviews followed a conversational format, transcripts were each analysed in entirety, rather than by question, and the transcripts were coded into relevant themes (e.g. types of behaviour). This process was deductive in that the authors had specific research areas that they were looking to examine that were reflected in the interview guide. However, data were coded inductively in that ultimately, participant perspectives drove the generation of the final themes. After a final list of codes was created, BM reviewed all transcripts a final time.

Excerpts of the interviews are reported in the *Results* to reflect participants’ thoughts and experiences within the research process. Quotations from participants are vital for providing support (Elo & Kyngäs 2008) and credibility (Côté & Turgeon 2005) for conclusions drawn during this study.

### Reflexivity statement

Identifying preconceived notions that researchers hold allows for better transparency within research and can offer additional context as to how researchers have approached their work (Holmes 2020). BM has previously worked in shelter dog behaviour in the USA, and she has an interest in addressing welfare questions in rescue dogs based on that experience. HZ is a veterinarian and a specialist in behavioural medicine. She has extensive experience consulting with dog owners both in general and behavioural practice. She has published in a range of areas in companion animal behaviour and training, but has not previously published qualitative studies. OB is a welfare scientist specialising in the development and refinement of measures used to assess the affective state of non-human animals. He has published research focused on behavioural and cognitive indicators of welfare in both owned dogs and those housed in a shelter environment using quantitative methodologies. BV works as an animal welfare scientist and draws heavily upon qualitative methods as part of her research programme; she has not published on companion animal welfare topics before, but spent several years as a volunteer in animal shelters in the USA prior to working in academia.

## Results

### Participant characteristics

Forty-five potential participants responded to the screening survey; three were excluded due to not meeting the study criteria or by failing to provide necessary information to determine inclusion. The remaining 42 individuals were invited to interview; of these, a total of 27 individuals were interviewed for this study. Interviews (n = 27) were conducted between March–April 2023 by BM over MS Teams and lasted between 16–58 min, with a mean interview length of 29 min.

Of those interviewed, five participants (19%) worked with pets in a professional capacity, most commonly listing their occupation as a dog walker or working in a rescue centre. Eighteen participants (66%) acquired dogs from organisations based in the UK, including both rescue centres and foster or volunteer-based rescue organisations, while the remaining nine had acquired their dogs from abroad (either from a rescue centre abroad or from a centre based in the UK that imported dogs for adoption; dogs were reported as originating predominantly from southern or eastern Europe [Cyprus, Romania, Greece, Macedonia, and Spain]).

### Themes

Participant interviews focused on six main themes: (1) time required for adjustment; (2) behavioural indicators during adjustment; (3) behaviours that indicate that adjustment has occurred; (4) factors that influenced adjustment; (5) changes to owners’ routine in response to their new pet; and (6) adopters’ concerns (Table 1).

**Table 1.** Themes, descriptions, and subthemes from participant interviews (n = 27) describing their experience with adopting a dog from a rescue shelter. N and % denote the number and proportion of participants drawing on specific themes/subthemes during their interviews. Due to rounding and as participants could mention more than one subtheme for the majority of the themes, values may exceed the number of interviewees and 100%

Theme	Description	Subthemes	n	%
1. Time required for adjustment*	The length of time that participants stated their dog required to adjust to the home	Reported adjustment within three months	8	30
		Reported adjustment between four to six months	11	41
		Reported adjustment as longer than seven months	3	11
		Reported not adjusted at the time of the interview	5	19
2. Behavioural indicators during adjustment	Behaviours that participants noted seeing while their dog adjusted to their home	Social contact	17	63
		Fear	17	63
		Rest and sleeping	15	56
		Overreaction to social and environmental stimuli	8	30
		House soiling	7	26
		Separation-related behaviours	7	26
		Eating (or lack thereof)	6	22
		Aggression	6	22
		Anxiety	5	19
		Presence or absence of exploratory behaviour	5	19
		Mouthy or inappropriate play	5	19
		Incidents of running away	4	15
		Resource guarding	2	7
3. Behaviours that indicate that adjustment has occurred	Behaviours that participants noted as an indication that their dog had adjusted or settled in to their home	Play behaviour	7	26
		Learning routine	5	19
		Greeting	3	11
		Tail wagging	3	11
4. Factors that influenced adjustment	Factors before or after adoption that participants attributed to causing a shorter or longer adjustment period.	Medical	6	22
		Resident dogs	4	15
		Prior life experiences	4	5
5. Changes to owner routine in response to their new pet	Aspects of their routine that participants noted needing to change as a result of acquiring their pet	Location or frequencies of walks	8	30
		Managing behaviour to visitors	3	11
6. Adopter concerns	Participants noting hesitations about their pet, owning their pet, or retaining their pet.	Making the right choice or meeting the dog’s needs	8	30

* For the purposes of reporting time to adjustment, participants who listed a range were reported as their dog having adjusted based on the lowest time they stated (e.g. a range of 3–4 months was placed at 3 months).

### Theme 1: Time required for adjustment

Most participants gave a time range when describing when they believed that their dog had adjusted to their home, rather than a specific number of weeks or months (e.g. 4–8 weeks, rather than 6 weeks). Some participants (4/27) were also uncertain as to how to assess whether their dog had adjusted. For example, one participant commented: *“It’s difficult to say because she was so good from day one…Yeah, that probably – the* [behaviour with the] *sofas probably stopped about that sort of four or five month mark. And the nipping has only recently stopped, so probably about 10–11 months. So yeah, basically it’s quite difficult to tell”* [P5].

Participants also mentioned that different behaviours they attributed to their dog being settled also corresponded to different time-frames. For example, some participants thought that their dog settled very quickly, but with a caveat, with one participant commenting, *“Sort of from day one. But then as time goes on, she’s more and more settled”* [P10]. Another said: *“Probably quite quickly, actually. He’s laid at my feet since the fourth day… So, I think quite early on, but he was four months old… he settled quite quickly”* [P13].

However, others felt that their dog took much longer to adjust. Overall, 30% (n = 8) of participants felt that their dog had adjusted within 3 months, 41% (n = 11) between 4–6 months, 11% (n = 3) more than 7 months, and 19% (n = 5) felt that their dog had still not settled at the time of interview (time in home ranged between 3–16 months, median time = 10 months).

### Theme 2: Behavioural indicators during adjustment

Participants named a wide range of behaviours used as indicators to assess their dog’s adjustment to their new home (see Table 1): social contact, fear, rest and sleeping, overreaction to social and environmental stimuli, house soiling, separation-related behaviours, eating (or lack thereof), aggression, anxiety, presence or absence of exploratory behaviour, mouthy or inappropriate play, incidents of running away, and resource guarding.

#### Social contact

One of the most commonly mentioned behaviours included the level of social contact that their new pet sought from their owner. Some (n = 10) described their dogs as seeking a lot of owner contact, often termed as ‘Velcro dogs’ as they were reported to frequently seek proximity to their caregiver. These dogs often appeared to be very attached to their owner right away:

*“… definitely those first few months we got a sense of her having like FOMO* [fear of missing out]*…it was with everything, so she followed us around the house for those first few months. We would put her bed in the kitchen when I was cooking, which I don’t have to do anymore. But it was obvious that she wanted to be in the room that you were in and if you left the room, she would, even if she was asleep, she would get up to go and see what you were doing”* [P5].

Other dogs quickly became affectionate towards their owners, but not necessarily to the same level seen in ‘Velcro’ dogs. While yet others noted attention-seeking from their dogs, with this often taking the form of behaviours owners considered less appropriate: *“He would just bark, bark, bark for attention because he hadn’t been trained before, so he needed to know the boundaries really”* [P7].

Other adopters had very little initial contact with their dogs (n = 6), as some were fearful of people and were thus avoidant of contact; for example, one participant shared, *“He used to drop, like if you touched him, he kind of froze, because he didn’t really know what to do… He wouldn’t go up, like come up to you willingly or anything like that”* [P16]. Over time, the amount of social contact moderated as the dog adjusted to the home, where ‘Velcro dogs’ became more independent of their owners, described by P4: *“If I…go out the room, she’ll come and see what I’m doing. But if I’m not doing anything interesting, she’ll go back and sit down in the room, whereas she never used to do that before unless I was in that room.”* Someone with an initially more fearful dog noted a similar moderation:

*“She would quite happily just lay on her own on a bed and just be left alone, whereas now she’ll crush me on the sofa, or she’ll lay across me, and she’s a lot more affectionate, like physical. She seeks a lot more tactile reassurance as well”* [P14].

#### Fear

Over half of participants (n = 17; 63%) described behavioural responses indicative of fear after adoption. One participant stated of his initially fearful dog: *“On his first night, he literally came in, he was scared, obviously uncertain, worried. He kind of pottered around in the garden a bit and then he kind of stayed in there in the back room”* [P20]. Another participant described their dog’s behaviour thus:

*“There was a lot of us just giving him space to approach us in his own time, and just a little bit of learning really about how we had to move around him, because any sort of sudden movements and he was just like, skittering away or cowering down on the floor”* [P1].

Several stimuli were noted to elicit these behaviours, including people (n = 6/27; 22%): *“I was told he was wary of people, and he is definitely more than wary, he’s terrified. They said he was fine with other dogs, and, again, he’s not”* [P2]. Some other participants also noted a fear of traffic (n = 3/27; 11%):

*“Outside, very nervous. Traffic, incredibly, really scared by it. We live on a side road, and I could barely take him to the top of the side road. So… we would go in the car and I would walk near the outside of a park”* [P25].

#### Rest and sleeping

Many (56%; n = 15) adopters also described how much their dog rested or, alternatively, displayed restlessness upon adoption. While some adopters noted a high level of rest and sleeping (22%; n = 6/27); for example, *“At the start we were fully expecting him to be wide awake all night, every night. He wasn’t. He just slept”* [P6], others reported that their dogs were restless and unable to sleep (33%; n = 9/27). Adopters who noted restlessness typically also mentioned that it was disruptive to their own sleep schedule: *“I was looking back the other day and at my sort of diary to getting an idea, and I didn’t realise how many times we were up in the night for the first few months”* [P11]. However, in both situations (i.e. either initially high or low levels of rest and sleeping), participants also noted that rest and sleeping tended to stabilise over time: *“She wouldn’t settle so much. She always wanted to play or be busy, and now she’s settles, and she knows she doesn’t go out all, every time I get up, and she will settle now and sleep the hours away and rest”* [P12].

#### Overreaction to social and environmental stimuli

Some adopters (30%; n = 8) also noted reactivity, which varyingly improved (11%; n = 3), remained the same (4%; n = 1), or became worse over time (15%; n = 4). Reactivity was most frequently seen in response to other dogs and people, but participants also mentioned their dogs being generally reactive to noise. Some also mentioned that reactivity to other dogs posed an additional problem due to the risk of their reactive dog being approached by others, particularly off-lead dogs:

*“He was very quick to react. So, if he heard a noise outside, he would leap off the sofa, charge to the back door and bark. And if the back door is open, charge around the garden, barking. He was very noise sensitive, that was probably one of the biggest changes is that he* [will] *sometimes do that, but it’s never as strong a reaction”* [P23].

#### House soiling

Owners (26%; n = 7) reported that house soiling occurred following adoption, with some sharing this was relatively minimal while others had dogs who needed more house-training. For example, one participant commented, *“He had a lot of accidents, the toileting accidents, and he did wee on the dog beds in particular, more than anything else”* [P23].

#### Separation-related behaviours

Adopters also had concerns about separation-related behaviours (26%; n = 7), although they were not always certain if their dog’s anxiety was due to a generalised source or a result of separation. For example, one interviewee shared:

*“I can trust him in the house for a few hours. Although I did mention that earlier as well – he’d suffered from, I don’t think it’s separation anxiety, or if it was just anxiety in general because again, we were told that they left him for a few hours in the house and he was fine. But when we did it, we came back and he chewed through the door frame”* [P26].

For some (19%; n = 5), the impact of their dogs’ separation-related behaviour problems was severe: *“She had a bit of separation anxiety, so I couldn’t leave the house. If I did…she was up on the counters, howling. There’s a glass door between the kitchen and the conservatory, and she’s trying to dig away out the glass door. So, it was like I was trapped in my house”* [P15].

#### Appetite

Participants (22%; n = 6) also used their dog’s level of interest in food as an indication of adjustment. When dogs immediately ate after entering the home, this was often seen as an indicator of comfort: *“He ate from the first day, which was a very good sign. Ate and drank things even if it was with his tail between his legs”* [P16]. However, several (11%; n = 3) noted that their dog was disinterested in eating: *“It was just a real struggle to work out what he did want to eat, and when he wanted to eat it, and how he wanted to eat it. I was quite worried because I didn’t think he was eating enough”* [P1], while others (11%; n = 3) were concerned that their dog’s appetite was boundless: *“very fast at eating his food, like he was never going to be fed again. I would say he’s a slowed down a little bit with that now”* [P18].

#### Aggression

While uncommon, instances of aggression towards humans, including that directed at members of the household or other people, was also noted by some participants (22%; n = 6):

*“He’s only done it a couple of times and…he used to growl at people… It’s more knowing that somebody could come up from nowhere and put their hand down and he might snap at them. I wouldn’t feel safe with a visitor coming in either, if he wasn’t on the lead”* [P23].

Some participants also expressed the fact that there were instances where their dog was less comfortable with a particular member of the household, leading to the dog growling or alarm barking at that person. Some found that aggressive behaviour was rare (4%; n = 1) or improved over time (4%; n = 1), while others said that they expected to be working with or managing it for a longer period (7%; n = 2).

#### Anxiety

A few participants also noted seeing anxious behaviours (19%; n = 5) in their dogs, which could occur as a general anxiety or related to a specific stimulus: *“I just felt sorry for him because when he’s anxious, he sits and he trembles and he wants to hide away behind things and underneath things. Nothing you can really do comforts them when they’re that far gone”* [P26]. Some also noted that their dogs began barking less as they settled in, which they related to anxiety:

*“When I got her at the shelter, she was labelled as very, very shouty, and yes, she is. She barks at everything. But as her anxiety disappears, and we play lots of games for confidence building, she’s like another dog. And she’s still quite shouty, but she no longer barks at absolutely everything”* [P10].

#### Presence and absence of exploratory behaviour

Interviewees (19%; n = 5) also mentioned their dog’s interest in their surroundings as another indicator of how their dog was adjusting. The degree of exploratory behaviour reported ranged from being hyperaware of surroundings (11%; n = 3) to ignoring them (7%; n = 2). An adopter of a hypervigilant dog noted that: “*We were also quite taken with how much he needed to be looking at things the whole time. He couldn’t just relax and like I said, that’s been a massive change”* [P6]. Conversely, an adopter of a dog who ignored her surroundings noted that:

*“We took her out for walks straight away, and she was very quiet and she let us walk her, she didn’t pull or anything on the lead….So, she was quite submissive, quite quiet…Because it was kind of the end of the summer, I just took her out lots for walks, and she soon became a lot more confident and sniffing around and looking, and was really interested in her surroundings”* [P4].

In both of these circumstances, participants noted an extreme behaviour from their new pet initially (either very high or very low), but eventually saw it change to a more moderate level of interest that was considered indicative of their pet adjusting to the home.

#### Mouthy or inappropriate play behaviour

Participants (19%; n = 5) noted mouthiness or otherwise inappropriate play or excitement behaviours. While these were generally mentioned as occurring shortly after adoption and often seen to improve over time, some experienced these as occurring over a longer-term. For example, one participant stated, *“I used to go and sit down and have a cuddle with my dogs, but he was very, very excited at that point and would jump up and start grabbing at my clothes”* [P23]. Another participant expressed a similar sentiment, saying, *“She also started doing this mouthing on us, where she’d put a mouth on you, not bite you, but it can be quite uncomfortable sometimes”* [P27].

#### Incidents of running away

A few participants also mentioned that their dog had initially tried to run away after they acquired them (15%; n = 4). One participant recalled of their newly adopted dog: *“In that first week, he escaped out of the front door at my in-laws and literally ran a mile across the other side of the town. Fortunately, we managed to get him back”* [P6]. This same adopter attributed the following scenario to the dog knowing where his home was, and wanting to be there:

*“Last summer he ran off… We went to look for him and as we came back home, he’d actually come home. So, he’s obviously been for a run, had a jolly nice time and then remembered where home was. And actually, we saw him on the road, at the front, and went to try and get him. He ran off and literally was waiting for me outside the front door. And I think that, so we’d probably had him about eight or nine months, then that was when I thought, actually, he knows where home is and he clearly wants to be with us, rather than not with us”* [P6].

#### Resource guarding

While it was not commonly reported, two participants (7%) also mentioned resource guarding and shared that they worked with this behaviour in training rather than expecting it to fully resolve:

*“He was quite bad with food. So as soon as I put food down, his head would be in a bowl and if you went near him, he’d growl a bit. But over time, I started to put in some obedience around waiting for meals and sort of a stay, and then he can eat when I say he can eat. Now he’s very good, and he’ll wait in his bed quite calmly but excited. But he won’t growl anymore”* [P21].

### Theme 3: Behaviours that indicate that adjustment has occurred

Participants noted several behaviours that indicated to them that their new pet had adjusted to their home, including showing play behaviour (26%; n = 7), learning their routine (19%; n = 5), greeting their owner when the owner came home (11%; n = 3), and wagging their tail for the first time (11%; n = 3).

#### Play behaviour

Participants (26%; n = 7) attributed the expression of play behaviours as an indication that their dog was adjusting to their home. Some mentioned this generally (*“She’s become a lot more playful”* [P8]), while others noted this specifically in relation to their dog’s behaviour with toys. One person noted that her dog never played in the shelter, but that behaviour came out after adjusting to the home, saying *“Looking back through her hand over stuff… they said she’d never play with toys…this dog flings toys in the air just for pure entertainment”* [P14]. Another described that their dog played with toys early on after arriving in their home, which they saw as an indicator that their dog was settling into their home: *“After a few hours, he started to play with toys, which we were surprised at because he seemed really shell-shocked”* [P20].

#### Learning routine

Participants (19%; n = 5) also noted that their dog began to seem more adjusted to the home when they began to show signs of knowing their routine. For example, one participant stated: *“In the morning, he knows his routine, he knows when his breakfast is, he knows when his tea is, he knows when we go out for a walk”* [P16].

#### Greeting

Some participants (11%; n = 3) said that they identified their dog was adjusting to their home when their dog greeted them; for example: *“He comes to the door now when I come home from work to greet me. But he only gets off the sofa for me… I don’t know how he knows it’s me coming through the door, but he does”* [P22].

#### Tail wagging

Finally, a few people (11%; n = 3) raised tail wagging as an indication that their dog had settled into their home: *“Once she started coming into the other rooms of the house with us, it’s just been a gradual improvement. But as I say, even up until Christmas, we noticed the wagging of the tail, but it’s all just been very slow”* [P12].

### Theme 4: Factors influencing adjustment

Participants identified different factors believed to influence how their new dog adjusted to their home, including medical factors (22%; n = 6), the presence of other pets (15%; n = 4), and their dog’s prior life experience (11%; n = 3).

#### Medical

With respect to medical factors, owners (22%; n = 6) attributed injuries, surgery, medical conditions, and pain as contributing to a longer adjustment period. Five out of these six participants mentioned the impact of various medical issues on adjustment, and three participants noted the impact of surgery on their dog’s adjustment. For example, one participant stated: “*I’d probably say longer than it should have because he had the medical issues. He had surgery about three months after we got him, so I think he started to settle, and then he had the surgery and it kind of set him back a bit”* [P17].

Some participants felt that, while medical factors may have made the adjustment period longer, they also noted that receiving medical treatment was crucial to their dog adjusting:

*“Turned out he had spinal problems. He went on medication and things got better, but I think there were still issues around pain, so they put him on different medication. They put him on gabapentin, and they put him on behaviour medication as well, so he went on fluoxetine, and it was probably within a day of going on the gabapentin, I would say he relaxed, he just seemed less scared and a happier dog”* [P23].

#### Resident dogs

The presence of other pets also impacted how participants (15%; n = 4) perceived their new dog’s adjustment, with several noting that their resident dog helped their new pet adjust to their home. For example, one participant commented, *“I think I feel like if we didn’t have another dog in the house, it would have been a lot slower process. We would have had to work a lot, a lot more to get him to settle in”* [P16]. While no one noted that their resident dog made the process more difficult, one participant attributed adopting a second dog as a part of the reason why their dog had not yet adjusted to their home.

#### Prior life experiences

Finally, some participants (15%; n = 4) attributed prior life experience, such as time spent in kennels (n = 2) or where the dog lived before coming into their home (n = 2), as influencing their dog’s adjustment to the home:

*“She was completely different because my last two dogs had come from a home environment, and especially the… very last one we had, the bond was sort of instant. She just wanted to be loved and she was up on the sofa and made herself at home straight away, whereas*, [dog]*, having come from obviously a puppy farm and then a kennel, she was much more cautious and reserved”* [P12].

### Theme 5: Changes to owner routine in response to their new dog

Most participants (96%; n = 26) did not find that it was particularly difficult to adjust their routine to accommodate their dog. Most frequently, participants (26%; n = 7) mentioned needing to adjust their routine to their dog’s energy level, typically by walking more:

*“It rapidly became apparent that he was gonna need a bit more exercise than I was expecting… The exercise became a longer-term change. Until he’s had his morning walk, he won’t settle. I’m usually taking him out for a walk within an hour of having got up. Otherwise he gets quite frustrated about it”* [P1].

Generally, participants did not find such routine changes to be overly difficult; rather, they expected their routines to change as a part of dog ownership. Some commented that these changes were made as a result of acquiring a dog with different energy needs or a drastically different personality to previously owned dogs, which would then necessitate changes to routines used previously: *“My other dog… He was a much easier, laid-back dog. Kind of you’ve gone from older, easier, laid-back dog to slightly unhinged, foreign, crazy dog, and I think that was a period of adjustment for us”* [P6].

Participants (11%; n = 3) also reported changes made to accommodate their dog’s fear or reactivity, for example, by finding more secluded places to walk: *“Some people he’s okay with. Other people, he doesn’t want to go near, and so then he gets frantic, so we can’t really walk him out on the street side, and we have to go to like quiet, secluded areas, open spaces that there’s not a lot of people”* [P17]. Similarly, others (n = 3; 11%) said that they needed to reduce visitor traffic or otherwise manage their dog’s behaviour towards visitors to their home to accommodate their dog’s response to people: *“I’ve never had an awful lot of visitors but I did sort of have to reduce that. I think that became obvious quite quickly that it was just very, very difficult to manage having visitors”* [P23].

### Theme 6: Adopter concerns

While at the time of interview only one participant expressed uncertainty that they would keep their dog, several (30%; n = 8) shared stories about having experienced doubts as to whether they had made the right choice in adopting their dog. Some expressed feeling guilt about having thoughts about returning their pet, or if they had done the right thing for their pet by adopting them. For example, one participant mentioned, *“I remember 8 months going, I can’t believe I’m thinking, I’m actually thinking it, never said it out loud, but actually thinking, I think this might be the time to, this just isn’t helping him and is it me”* [P7].

Participants also indicated that their thoughts about having made a mistake often oscillated, e.g. *“So, one day I want to send her back and the next day I think, ‘Oh can I? Can’t really do that now’”* [P15]. Others expressed that their relationship with their pet was not necessarily what they expected initially, or that it took more time for a meaningful bond to develop:

*“I’d say the first year or so, there were times when I definitely regretted getting him, and purely because of some of the extra levels of, or layers of things we’re having to include with him. Particularly because he is a flight risk, and particularly because he is so reactive. And I did find myself, not hating him, but…not being able to have the relationship you want with a pet”* [P6].

## Discussion

This study explored adopter experiences during the post-acquisition adjustment period of rehomed dogs and focused on people whose dog had lived in their homes for a minimum of three months. We found that, whilst there was individual variation amongst owners in their estimates of how long it took for their dogs to adjust to the home, owners’ experiences here suggest that the adjustment period likely lasts beyond three months for many dogs. We were also able to identify behaviours that owners may typically see when acquiring a new rescue dog, as well as those behaviours likely to indicate that a dog has adjusted to their new home.

## Duration of adjustment

One of the prominent findings of this study was that the adjustment period, as perceived by the owners interviewed, appears to be longer than current adoption literature suggests. Relatively few participants in our study (8/27 of total participants) gave estimates that aligned with the two week (BARCS undated), four-to-six week (Wolfe 2019), and three-month (Aldred 2022) time-frames suggested in the adoption literature. This finding suggests that while some dogs may adjust relatively quickly, many may take significantly longer to fully adjust than previously postulated. This aligns with more recent findings that changes continued to occur in the six months following adoption (e.g. Van der Laan *et al.* 2022; Bohland *et al.* 2023). We propose that while adjustment times may vary among individual dogs, owners should be made aware that a prolonged period of adjustment could be required, and so rehoming organisations need to be prepared to support owners throughout this time.

Some adopters noted that medical factors influenced their dog’s adjustment. While aspects like pain management typically helped their pet adjust, adopters also noted that surgery or some medical diagnoses seemed to make it more difficult for their dog to adjust. This is in line with prior evidence that dogs exhibit behavioural changes (e.g. activity level, play, and contact seeking) following surgery (Väisänen *et al.* 2004). This is an area that may therefore merit further research to determine how routine surgeries, such as spaying and neutering, may impact newly adopted dogs, and how these effects can be mitigated to avoid extending the adjustment period.

Another factor that participants mentioned as influencing the adjustment period was the occurrence or length of kennelling prior to adoption. As kennelling can produce a physiological stress response and cause behavioural changes in dogs (e.g. Rooney *et al.* 2007), this indicates that kennelling and potentially other life experiences may play a role in how dogs adjust to a new home. We are aware that some factors, for example, gradual habituation to a kennel, can mitigate these stressors (Rooney *et al.* 2007), but more work on the reduction of kennel-related stress would be beneficial.

### Behavioural extremes

Participants often mentioned seeing extreme behaviours (either initially very high or very low levels of the same behaviour) that then moderated as their dogs adjusted to the home. These extreme behavioural responses could potentially serve as a proxy measure for the degree to which a dog is coping, and thus prompt additional interventions to better support the transition for dog and owner. For instance, adjustments in social contact between dog and owner was one of the behaviours seen during the adjustment period, in alignment with recent work indicating that shelter dog attachment and attention-seeking scores on the C-BARQ decreased over 180 days following adoption (Bohland *et al.* 2023). While Bohland *et al.* (2023) noted that this behaviour could be viewed as either a positive or negative depending on the owner’s preferences, participants in our study who owned ‘Velcro dogs’ found their dog’s moderation of behaviour to be positive and were typically happy with their dog’s increased independence. Diesel *et al.* (2008) found that a commonly cited reason (15% of returns) for returns was that dogs needed more attention than owners had anticipated. Given that many returns occur in the initial weeks post-adoption (Diesel *et al.* 2008), clarifying to owners that this behaviour may moderate over time may help reduce premature returns.

However, our research also found individual differences amongst the dogs. In direct contrast to the ‘Velcro dogs’, other dogs were initially avoidant of their owners and became more affectionate over time. While tools like the C-BARQ, frequently used in behavioural assessment of dogs, can identify population differences in studies such as those cited, many aspects of dog ownership require a more individualistic approach. For example, Blackwell *et al.* (2006) found that separation-related behaviours benefit from a tailored approach. As our results suggest that some dogs may show opposite behavioural extremes with respect to social contact, counselling owners in their expectations and understanding of the potential for behavioural moderation over time may be of benefit to both animals and adopters, potentially facilitating successful long-term placements.

In our study, although several participants noted that their dog rested or slept a lot initially and became more energetic over time, others noted that their new pet initially exhibited restlessness, sometimes for an extended period. Research has previously shown that sleep patterns can be a reliable indicator of welfare; in shelter dogs, increased daytime resting behaviour is associated with other positive welfare outcomes such as a more positive judgement bias and less repetitive behaviour (Owczarczak-Garstecka & Burman 2016). Similarly, shelter dogs have higher night-time activity and disrupted rest compared to those in a home (van der Laan *et al.* 2023) or in fostered environments (Gunter *et al.* 2019), with shelter dogs also showing more night-time activity in their first days in the shelter (van der Laan *et al.* 2023). Van der Laan *et al.* (2023) also showed that, over time, dogs appeared to partly acclimate their resting patterns to shelter schedules. It is therefore possible that the restlessness experienced by some adopters in our study is due to an environmental change post-adoption resulting in a disruption of sleep patterns. As restlessness is more likely to be disruptive to the adopter’s schedule and ability to sleep, it is important to understand more about what may lead to this behaviour occurring and how it can be addressed to improve adoption success.

### Behaviours indicative of concern

It may not be possible to completely eliminate fear responses shown by dogs in a changing environment, particularly when considering individual differences in confidence or resilience in dogs (Tiira 2019). However, as over half of participants in our study saw this response, it presents the opportunity to work towards reducing fear responses and thus improving welfare. Furthermore, as fear responses may also be comorbid with or a precursor to other behaviours like aggression (Willen *et al.* 2019), better understanding how post-adoption fear responses can be mitigated may also be beneficial to improving dog outcomes. Willen *et al.* (2019) found that enrichment was beneficial in reducing fear responses in shelter dogs, so pre- and post-adoption strategies involving enrichment could be examined to assess if they reduce the occurrence of fear. Determining if there are additional factors that can pre-emptively reduce the fear response, as well as identifying the best response to fearful dogs during the adjustment process, will help owners to better understand and support their dogs.

Overreactivity to stimuli may occur for multiple reasons, including as an overt response to excitement, fear, or frustration. While research has linked types of reactivity to aggressive responses, this is not always the case; dogs may also react to unexpected stimuli in an overreactive, but non-aggressive, manner (Arata *et al.* 2014). Better understanding of reactivity and differentiating these responses would be beneficial for adopters to address their dog’s specific response and the underlying causes. Whilst the reasons for reactivity can vary, it is important to consider the welfare of reactive dogs. Reactivity correlates with physiological signs of stress; prior research has found that cortisol increases in dogs in response to barks that indicate an intruder and those that indicate play, and higher reactivity was also associated with elevated cortisol levels two weeks later (Siniscalchi *et al.* 2013). Identifying if reactivity occurs during the adjustment process and when it could be a welfare indicator may be beneficial in helping adopters to obtain appropriate assistance for their pet.

Many adopters expressed concern about separation-related behaviour problems, and some adopters also expected to see separation-related behaviours improve or dissipate as their dog adjusted. Prior research found that separation-related behaviours decreased between 90 and 180 days following adoption (Bohland *et al.* 2023). This indicates that some separation-related behaviours may naturally decline in the post-adoption period. While this was also expected by some adopters in our study, this was not always the case; some noted that they felt that their pet’s separation-related behaviours became worse, or did not improve as their dog remained in the home. The occurrence of separation-related behaviours can put dogs at risk for re-relinquishment (Hawes *et al.* 2020), and some evidence suggests that dogs acquired from shelter and rescue organisations may be more likely to develop separation-related behaviour problems (Flannigan & Dodman 2001). Being able to identify what changes in separation-related behaviours can generally be expected, and what is indicative of adopters needing additional support, as well as being able to connect them with appropriate resources may be important for supporting adopters faced with these issues.

Participants in our study also identified eating and lack of eating as an indication of their dog’s comfort level. While refusing to eat may be an indication of stress or poor welfare (Kartashova *et al.* 2021), it can also be associated with medical issues (Bourgeois *et al.* 2006). Owners identify reduced appetite as an indication of stress (Mariti *et al.* 2012), so being able to better prepare owners for this risk during the adjustment period is likely important. However, adopters should also be aware that this behaviour could result from an underlying medical condition, and thus they may need to follow up with a veterinary professional regarding potential medical concerns.

While instances of aggressive behaviour were not commonly mentioned in this study, they did occur. Bohland *et al.* (2023) found evidence that stranger-directed aggression increased over the first 180 days in the home. Our study partially aligns with this, in finding that some adopters expected to manage their pet’s behaviour long-term due to this behaviour. However, some adopters in our study also found that the aggressive behaviour decreased. The occurrence of aggression, even if rare, is important as it can have risks for people and other animals around the dog. Aggression can be a reason for relinquishment (Hawes *et al.* 2020), as well as increasing the risk of it (Wells & Hepper 2000). A better understanding of aggressive responses during the adjustment period may be useful in preventing situations of risk and establishing when adopters should seek out additional support.

### Positive behavioural indicators

Participants in our study described positive behavioural indications of adjustment, including engaging in play behaviour, learning a new routine, greeting their owner, and tail wagging. The presence and absence of play behaviour can be used as a welfare indicator, although the relationship may be nuanced (Held & Spinka 2011). Kennelled dogs spend relatively little time playing, but their welfare can be improved with the provision of toys, likely due to the subsequent decrease in inactivity (Wells 2004). This indicates that adopters may be able to improve the welfare of their dogs by providing toys, and that dogs’ willingness to play may be an accurate indication of adjustment. Further research on the emergence of this potentially important behaviour after adoption is therefore required.

The finding that owners notice dogs learning household routines is an interesting one, and to our knowledge has not previously been reported. A household routine is something that is predictable to a dog and as predictability has previously been linked to reduced stress levels in animals (Bassett & Buchanan-Smith 2007), routines may benefit welfare. Noticing dogs learning routines may have potential as a positive welfare indicator, beyond simple adjustment to the new environment. In addition, it may be an area to explore as an intervention; for example, Raudies *et al*. (2021) and Amat *et al.* (2014) have both suggested ways in which predictability could benefit welfare and behaviours indicative of negative affective state both within the shelter and within the home.

Finally, tail wagging is generally considered to be a positively valenced behavioural sign in dogs (e.g. McGowan *et al.* 2014; Travain *et al.* 2016), and was reported by owners in our study. However, tail wagging can also communicate negative arousal (Leonetti *et al.* 2024). For this reason, before utilising ‘tail wag’ as a positive welfare indicator, tail posture and other body language seen concurrently should be assessed to determine if it truly is a positive indication of adjustment. Our study provides evidence that these are key behaviours that owners see emerge as they develop a relationship with their new dog, and so have the potential to be informative indicators of adjustment.

### Adopter concerns

Adopting a dog is often an adjustment for the owner and other members of the household as well as for the animal. Many adopters struggle when making decisions about returning their pet (Thumpkin *et al.* 2024) and may not want to adopt again (Shore 2005). While all participants in our study still owned their dog when interviewed, some participants did mention having hesitations about the pet they acquired, highlighting that adopting and retaining an animal can be difficult. Most dogs are returned to shelters for behavioural or personal reasons (Diesel *et al.* 2010; Hawes *et al.* 2020; Powell *et al.* 2021), aligning with some of the reasons given by adopters in our study. Further investigation of factors that predict when adopters are likely to question keeping their pet and why some adopters retain their pets and others do not, may be a useful next step to help rescues implement strategies and connect adopters to the resources they need.

### Study limitations

It is likely that the participants in this study may have had a particular interest in both rescue dogs and dog behaviour, and so their experiences may not be reflective of all dog adopters. Furthermore, there may be differences in adjustment based on dog-related factors, including age, breed, and differences between rescue organisations. While some participants mentioned these during interviews, demographic information on the dogs was not collected, and it was beyond the scope of this study to characterise the impacts of these factors. Additionally, as demographic data were not collected regarding the human participants in this study, there may be a variety of owner characteristics that may also impact adjustment that would be of interest to investigate in future work. This study also relied upon owner assessments of when their dog adjusted to the home and how owners perceived that process. Since owner reports of adjustment have not yet been validated, it is possible that these owner estimates are inaccurate. However, as adjustment is a process experienced by both dog and owner, having a better understanding of how owners as primary caregivers perceive this period remains important.

### Animal welfare implications

This study highlights the variation amongst rescue dog owners in their perceptions of, and experiences with, the post-adoption adjustment period. We suggest that for many dogs, the adjustment period may last longer than estimates given by adoption organisations. The dog owners interviewed in this study described several key dog behaviours (e.g. activity and resting behaviour, social interaction, fear) that should be considered when supporting adopters and their dogs through the adjustment period. Owners reported that certain behavioural extremes became more moderated over time, which suggests that behavioural moderation may be an additional useful indicator of adjustment having taken place. Other important behavioural indicators of positive welfare (e.g. play, tail wagging) appear to emerge only once a dog has adjusted to its new environment. Collectively, these results suggest that newly adopted dogs may employ different ‘strategies’ in response to the challenge of coping with, and adapting to, environmental change coinciding with adoption into a new home. When considering how welfare can be improved in newly adopted rescue dogs alongside managing owner expectations, understanding the implications and changes that occur during this transitionary process is important. A better understanding of this process may also help improve owner experiences, retention rates, and allow rescue organisations to provide better post-adoption support.

## Supporting information

Moyer et al. supplementary material Moyer et al. supplementary material

## Supplementary material

The supplementary material for this article can be found at http://doi.org/10.1017/awf.2025.4.
