# Supplementary material for: A qualitative exploration of owner experiences following dog adoption
Source: Anim Welf. 2025 Feb 10;34:e9. doi: 10.1017/awf.2025.4 (PMC11810508; doi:10.1017/awf.2025.4)
Supplement: Moyer et al. supplementary material [file S0962728625000041sup001.pdf]

A qualitative exploration of owner experiences following dog adoption

Bethany Moyer https://orcid.org/0009-0002-5551-487X, Helen Zulch, Beth Ventura https://orcid.org/0000-0001-9476-6901, Oliver Burman

Animal Behaviour, Cognition, and Welfare Research Group, Department of Life Sciences, University of Lincoln, Brayford Pool Campus, Lincoln LN6 7DL, UK

Author for correspondence: Bethany Moyer, email: 26229179@students.lincoln.ac.uk

Supplementary material

Interview Guide

Acclimation

1. Can you tell me what you remember about the first day/night after bringing your dog home?
   - What were you expecting?
   - Did anything happen that you didn't expect?
   - Can you tell me about the first week?

2. Did you need to make any changes to your daily routine after adopting your dog? Specific changes when you first brought your dog home?
   - Long term changes to accommodate your dog?
   - How do you feel about those changes?

2. How did you identify when your dog had settled into your home?
   - How long did it take to get to fully settled?

3. Do you feel like your dog's personality has changed or become more obvious since he/she first came home?
    - If "yes"; please can you briefly describe the changes you have noticed
4. What challenges or concerns did you have in the first few weeks after bringing your dog home?
    - Related to the dog?
    - Related to other factors?
5. What positive experiences did you have in the first few weeks after bringing your dog home? Was there anything positive you didn't expect?
    - Related to the dog?
    - Related to other factors in your life?

Relationship/Attachment

6. Do you feel your relationship with your dog has changed since you got him/her?
    - In what way and over what time scale?
    - From the perspective of the owner
    - Do you have the relationship with your dog that you hoped for?
        i. If "no"; can you explain how what you have differs from your expectations
7. Has your dog's relationship with you changed since you got him/her?
    - In what way and over what time scale?
    - From the perspective of the dog- has your dog's interaction with you/perception of you changed?

Behaviour

8. Can you describe your dog's behaviour during the first few weeks after being brought home? What changes, if any, did you see over time?
9. Did you see any behaviour from your dog that ever concerned you?
    - When did it occur?
    - Did you seek help from anyone about that behaviour or for other assistance?
        - Why/Why not?
        - If yes, who?
    - Did the behaviour resolve?
        - How long did it take to resolve?
        - What did you do to help resolve it?

Intervention/Support

10. What support did you want or need from the rescue or other animal professionals, especially shortly after you brought your dog home?
    - Do you feel like you had that support?
    - What contact, if any, did you have with the shelter / rescue organisation after your dog's adoption?
11. Did you feel like you had the knowledge and tools you needed (from the rescue, trainers, etc.) to be set up for success?
    - If Yes: Why was this?
    - If No: What do you wish you had known, or had access to?
12. How likely would you be to adopt another rescue dog in the future?
    - What about your experience most made you feel this way?

13. Is there anything you would do differently, if you were to adopt another dog in the future?
    - Why/Why not?
    - What would you want to change?
14. What advice would you give someone else, if they were adopting their first dog from a rescue / rehoming organisation? – *possible neutral question*

Later Studies – Possible neutral questions

15. In our future research, we hope to investigate more deeply into adopted dog behaviour and welfare. If you were to consider participating in our future studies, how much time would you be willing to commit?
    - Daily, weekly, or monthly
16. What sort of participation would you be prepared to engage with, if any?
    - Have someone visit your home to observe your dog in its home environment or to conduct short behavioural tests with your dog (e.g. their response to meeting a new person)?
    - Submit videos of your dog?
    - Conduct short behavioural tests with your dog yourself?
    - Complete short surveys?

Final Thoughts

17. Is there anything you would like to add that we haven't talked about?
